# Low Vitamin D Status and Suicide: A Case-Control Study of Active Duty Military Service Members

**DOI:** 10.1371/journal.pone.0051543

**Published:** 2013-01-04

**Authors:** John C. Umhau, David T. George, Robert P. Heaney, Michael D. Lewis, Robert J. Ursano, Markus Heilig, Joseph R. Hibbeln, Melanie L. Schwandt

**Affiliations:** 1 National Institute on Alcohol Abuse and Alcoholism, National Institutes of Health, Bethesda, Maryland, United States of America; 2 Osteoporosis Research Center, Creighton University Medical Center, Omaha, Nebraska, United States of America; 3 Department of Preventive Medicine, Uniformed Services University of the Health Sciences, Bethesda, Maryland; 4 Department of Psychiatry, Uniformed Services University of the Health Sciences, Bethesda, Maryland, United States of America; Chiba University Center for Forensic Mental Health, Japan

## Abstract

**Objective:**

Considering that epidemiological studies show that suicide rates in many countries are highest in the spring when vitamin D status is lowest, and that low vitamin D status can affect brain function, we sought to evaluate if a low level of 25-hydroxyvitamin D [25(OH)D] could be a predisposing factor for suicide.

**Method:**

We conducted a prospective, nested, case-control study using serum samples stored in the Department of Defense Serum Repository. Participants were previously deployed active duty US military personnel (2002–2008) who had a recent archived serum sample available for analysis. Vitamin D status was estimated by measuring 25(OH) D levels in serum samples drawn within 24 months of the suicide. Each verified suicide case (n = 495) was matched to a control (n = 495) by rank, age and sex. We calculated odds ratio of suicide associated with categorical levels (octiles) of 25(OH) D, adjusted by season of serum collection.

**Findings:**

More than 30% of all subjects had 25(OH)D values below 20 ng/mL. Although mean serum 25(OH)D concentrations did not differ between suicide cases and controls, risk estimates indicated that subjects in the lowest octile of season-adjusted 25(OH)D (<15.5 ng/mL) had the highest risk of suicide, with subjects in the subsequent higher octiles showing approximately the same level of decreased risk (combined odds ratio compared to lowest octile  = 0.49; 95% C.I.: 0.315–0.768).

**Conclusions:**

Low vitamin D status is common in active duty service members. The lowest 25(OH)D levels are associated with an increased risk for suicide. Future studies could determine if additional sunlight exposure and vitamin D supplementation might reduce suicide by increasing 25(OH) D levels.

## Introduction

Suicide is a global health concern and ranks as one of the leading causes of death worldwide. Among the United States military, suicide has become a critical issue. The increased risk of suicide in areas with less sun exposure, and during the spring when 25-hydroxyvitamin D [25(OH)D] levels are at their lowest [1]–[3], suggests that some seasonally determined factor could increase the risk for suicide. Seasonal changes in sunlight exposure profoundly affect the amount of UV-B light which penetrates the epidermis to stimulate the production of pre-vitamin D and the subsequent levels of 25(OH)D [3], [4]. It is this metabolite of vitamin D that is measured to obtain an index of an individual's vitamin D status. Although vitamin D can be obtained from the diet, more than 90% is produced by the effect of sunlight, resulting in a sometimes substantial seasonal variation in the circulating levels of 25(OH)D. As needed, 25(OH)D is 1-α-hydroxylated in the brain and other tissues to produce an active form, 1,25-dihydroxyvitamin D, which serves as the ligand for vitamin D receptors found in both the cell membrane and nucleus.

There is increasing evidence that vitamin D influences brain function [5]–[9]. Transcription of more than 1,000 genes is known to be under the control of vitamin D, potentially contributing to neurotrophic and neuroprotective effects which could influence suicidal behavior [3], [5]. These transcriptional effects are mediated by nuclear vitamin D receptors (VDR) found in many areas of the brain, and VDR gene variants are associated with cognitive function and depressive symptoms [9]. Although suicide is not always accompanied by depressive symptoms, several recent large epidemiological studies support an association between Vitamin D and depression [10]–[12].

To evaluate the possibility that lower vitamin D status could be associated with increased risk of suicide, we examined the 25(OH)D levels in archived serum obtained from service members who subsequently died by suicide and compared them to matched controls. We predicted that suicide risk would follow a pattern similar to what is often seen with other nutrient deficiencies, i.e., an increased risk would occur below some threshold value of vitamin D status.

## Methods

### Study Population

The study population included active duty service members from the United States military who had been deployed and for whom blood serum was available from the Defense Medical Surveillance System (DMSS), Armed Forces Health Surveillance Center (AFHSC). From this population, all officially verified suicides occurring between 2002 and 2008 who had blood sampled within 24 months of death were included as cases (n = 495). Suicides were considered official by the Medical Mortality Registry after detailed investigative review and confirmation by the Armed Forces Institute of Pathology. Control subjects were randomly selected from the same population by the AFHSC. Controls were matched by age (+/− six months), sex, and rank. In the military, rank reflects education, income and other socioeconomic factors. The controls were also matched for blood serum drawn within 12 months of the serum draw of their matched case to minimize variability due to temporal changes in the military environment. This is a subset of a previously described population [13]. The date of blood collection and suicide was provided from AFHSC in two month intervals to preserve anonymity of the suicide casualties. Mental health related ICD-9CM discharge diagnosis codes from all available standardized reports were provided from AFHSC and used to determine if the subject had any history of depressive diagnosis. A subsample of the suicide cases (n = 300) also had data from a Post-deployment Health Assessment (DD Form 2796), which includes items related to experiences during deployment as well as to post-deployment mental health.

### Ethics Statement

This study was approved by the institutional review board of The Uniformed Services University (FWA00001628; DOD assurance P60001) May 8, 2009, human subjects research protocol HU873B-01. All archived data and blood serum were re-coded to eliminate personally identifiable information before release to the investigators. Because of the importance of preserving the anonymity of the individuals who died by suicide and therefore could not provide consent, special care was taken to eliminate any details of the suicide which might allow identification.

### Laboratory Analysis

Serum sample levels of 25(OH)D, a principal storage form of vitamin D, were determined by enzyme immunoassay (Immunodiagnostic Systems Inc, Fountain Hills, AZ), with a sensitivity of 2 ng/mL and intra- and interassay coefficients of variation of 5.3% and 4.6%, respectively.

### Statistical Analysis

Baseline characteristics of the suicide cases and controls were analyzed with descriptive statistics and are reported in Table 1. Because of the anticipated seasonal variation in 25(OH)D concentrations and because we were unable to match subjects by date of blood draw, we adjusted the 25(OH)D concentrations for seasonal variation using a sine wave algorithm [14], i.e.,
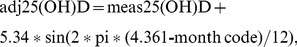
(1)Where adj25(OH)D is the value of 25(OH)D adjusted for season of draw, “sin” and “pi” have their usual mathematical meaning, and the month code was as follows: February/March  = 2, April/May  = 4, June/July  = 6, August/September  = 8, October/November  = 10, and December/January  = 12. This adjustment completely removes variation due to season of blood draw [14]. The parameters of the algorithm were determined empirically by fitting Eq. 1 to the 25(OH)D values of the control subjects. After adjusting for seasonal variation in this manner, we calculated octiles of 25(OH)D based on the distribution of the control subjects (see footnote to Table 2), and used the octile boundaries to assign both cases and controls to each octile category [15]. Additionally, the risk estimates (odds ratios, OR), were fitted to a model typical for nutrients (which exhibit change in effect only over the low end of the intake range, e.g., iron intake and hemoglobin concentration), as follows:
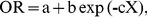
(2)Where the parameter a represents the estimate of the steady state value above the response threshold, b, the increment (decrement) due to the altered vitamin D status, and c, the exponential parameter describing the rapidity of the approach to steady state.

**Table 1:** Mean plasma 25(OH)D levels and baseline characteristics for cases and controls.

Characteristic	Suicide Cases (n = 495)	Controls (n = 495)
25-hydroxyvitamin D^1,2^ (SEM), g/ml	24.5 (0.5)	24.8 (0.5)
Season of blood draw (number of subjects)^3^		
December/January	64	86
February/March	103	79
April/May	79	88
June/July	85	82
August/September	73	72
October/November	91	88
Sex		
Males	467	467
Females	19	19
Age at Suicide (SEM), yr	28.5 (0.3)	
Ethnicity		
Asian/Pacific Islander	24	21
Black	63	79
Hispanic	54	62
Native American	12	4
White	326	314
Other/Unknown	16	15
Branch of Service		
Army	249	238
Air Force	92	96
Marine Corps	69	86
Navy	85	75
Grade		
Enlisted	454	454
Commissioned Officer	36	36
Warrant Officer	5	5
Months Deployed^4^ (SEM), months	9.6 (0.3)	11.0 (0.3)
History of Depressive Diagnosis^5^	109	67

1 To convert to nanomoles per liter, multiply by 2.496.

2 There was no significant difference between suicide cases and matched controls in 25(OH)D levels, either not adjusted for season of blood sample collection (t_494_  = 0.41, p = 0.68), or adjusted for season of blood sample collection using a waveform algorithm (t_494_ = 0.43, p = 0.67).

3 Only bimonthly categories were available for season of blood draw, as the dates of both blood collection and of suicide were provided from AFHSC in two month intervals to preserve anonymity of the suicide casualties.

4 Significant difference between suicide cases and matched controls based on a paired t-test (t_494_ = 3.68, p = 0.0003).

5 Significant difference in frequency between cases and matched controls (X^2^ = 12.2, p = 0.0005).

**Table 2:** Results of the conditional logistic regression analysis including covariates.

Parameter	No. Cases/ Controls	DF	Estimate	Standard Error	Wald Chi-Square	p value	Odds Ratio (95% Conf. Limits)
Vitamin D Octile 1^1^	84/61		reference				
Vitamin D Octile 2	58/62	1	−0.71	0.28	6.25	0.01	0.49 (0.28, 0.86)
Vitamin D Octile 3	56/63	1	−0.79	0.29	7.22	0.007	0.45 (0.26, 0.81)
Vitamin D Octile 4	31/60	1	−1.51	0.33	20.42	<0.0001	0.22 (0.12, 0.43)
Vitamin D Octile 5	55/62	1	−0.80	0.30	7.19	0.007	0.45 (0.25, 0.81)
Vitamin D Octile 6	85/65	1	−0.32	0.29	1.25	0.26	0.73 (0.41, 1.27)
Vitamin D Octile 7	67/59	1	−0.56	0.29	3.73	0.05	0.57 (0.32, 1.01)
Vitamin D Octile 8	59/63	1	−0.73	0.30	5.81	0.02	0.48 (0.27, 0.87)
Ethnicity^2^		1	−0.58	0.23	6.43	0.01	0.56 (0.36, 0.88)
Months Deployed		1	−0.04	0.01	12.55	0.0004	0.96 (0.94, 0.98)
Depression Diagnosis	109/67	1	0.69	0.18	14.16	0.0002	1.98 (1.39, 2.84)

1 Octiles were calculated from waveform adjusted 25(OH)D levels based on the distribution of the control subjects. Cutoff 25-hydroxyvitamin D values were as follows. octile 1: ≤15.5; octile 2: 15.6–18.8; octile 3: 18.9–21.2; octile 4: 21.3–22.9; octile 5: 23.0–25.4; octile 6: 25.5–29.2; octile 7: 29.3–36.0; octile 8: >36.0.

2 Odds ratio based on the comparison of African American vs. Non-African American.

Because we postulated that optimal brain function might require a threshold level of 25(OH)D, we used conditional logistic regression analyses to estimate the association between octiles of 25(OH)D concentrations and risk for suicide. Preliminary analyses were conducted to evaluate the potential contribution of a number of covariates to the regression model, including baseline characteristics (sex, ethnicity, branch of service, rank, number of months deployed), history of a depressive diagnosis, and items from the DD 2796 form. Because DD2796 items were only available for 300 of the 495 suicide cases, we first evaluated the potential contribution of these variables in this subsample of cases and their controls. The items evaluated included questions related to experiences during deployment (i.e., danger of being killed, witnessing death, or engaged in direct combat) and questions related to mental health in the month preceding completion of the form (feeling detached, down, or depressed, having thoughts about hurting oneself, feeling little interest, feeling loss of control, experiencing nightmares, being constantly on guard, intention to seek help for mental health related problems, and receiving a referral for mental health). None of these variables were significantly related to suicide risk. Therefore, subsequent analyses were conducted using the larger sample of all cases (n = 495 with recent deployments) and their matched controls. Preliminary associations with suicide risk were found for race (African-American vs. non African American), history of depressive diagnosis (yes/no), and the number of months deployed, and thus were included as covariates in the final model used to evaluate the association of adj25(OH)D and suicide risk. Odds ratios and 95% confidence intervals were calculated based on the conditional logistic regression model, with the lowest octile (octile 1) as the reference.

## Results

Mean serum 25(OH)D concentrations (either unadjusted or adjusted for season of serum collection) did not differ between suicide cases and controls (Table 1). Furthermore, 35.0% (173/495) of controls and 34.6% (171/495) of suicide cases had levels less than 20 ng/ml when unadjusted for season. When adjusted for season of serum collection, 31.1% (154/495) of controls and 33.5% (166/495) of suicide cases had levels less than 20 ng/ml. 25(OH)D was lower in African Americans, and for this ethnic group, 86.1% (68/79) of controls and 81.0% (51/63) of suicide cases had levels less than 20 ng/ml; when adjusted for season, 78.5% (62/79) of controls and 79.4% (50/63) of suicide cases had levels less than 20 ng/ml. Consistent with the biology of vitamin D, we found a marked seasonal variation in prevalence of deficiency. For example, among subjects sampled in February/March, 64.6% (51/79) of controls and 62.1% (64/103) of cases had levels less than 20 ng/ml, compared to 13.4% (11/82) of controls and 17.6% (15/85) of cases sampled in June/July. Suicide cases and controls differed on the number of months deployed, with control subjects having a slightly higher length of deployment (Table 1).

**Figure 1:**
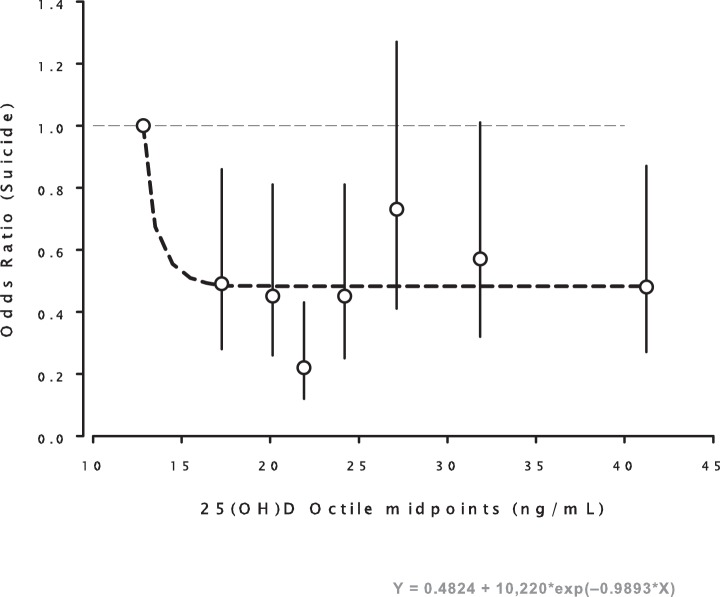
Plot of the computed odds ratios (OR) for the 8 octiles of 25(OH)D concentration, locating each at the mid-point of the respective octiles. The vertical lines at each point represent the 95% confidence intervals for each odds ratio, (see Table 2, footnote 1). The dashed line is the least squares fit of the 8 values to the equation: OR = 0.48 + 10,220 exp (−0.9893X), which is the expression best suited to describe nutrient effect as a function of intake. As is visually apparent, the regression line falls within the confidence limits for 6 of the 7 octiles above the first. (Copyright, Robert P. Heaney, 2012. Used with permission.).

**Figure 2:**
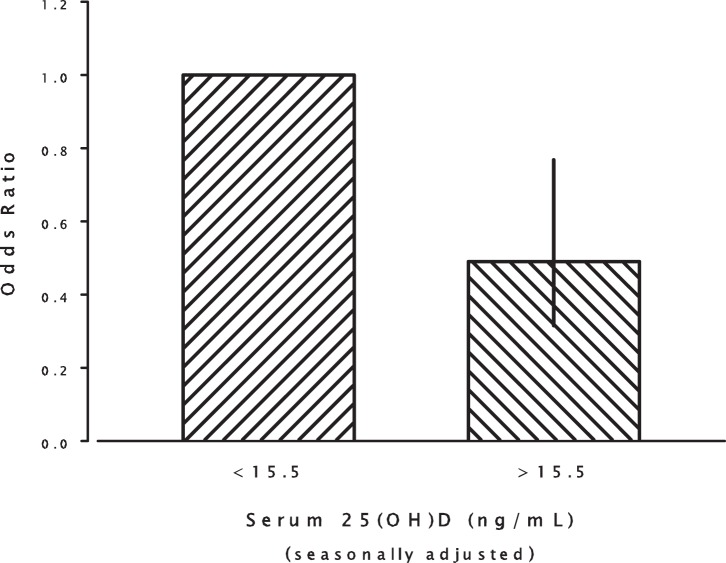
Plot of the odds ratio for suicide for the top seven octiles, relative to the lowest octile. OR = 0.49 (95% C.I. 0.315–0.768).

When controlling for season of serum collection, race (African-American vs. non African American), history of depressive diagnosis, and length of deployment, we found a statistically significant association between 25(OH)D concentrations and suicide risk, such that subjects with higher concentrations of 25(OH)D displayed a decreased risk for suicide compared to subjects in the lowest octile (Table 2). Fig. 1 displays odds ratios by octile and adds a least squares regression line fitted to the data using Eq. 2, a model that describes the typical relationship of nutrient intake and related outcomes [16]. The top seven octile risk estimates did not differ from one another, and when those risks were aggregated, the combined relative risk for octiles 2–8 was 0.49 (C.I.: 0.315– 0.768) (Fig. 2).

Risk of suicide was also associated with a previous diagnosis of a depressive disorder (OR  = 1.98, 95% confidence interval  = 1.39 to 2.84; Table 2). However, when we conducted a logistic regression analysis in this study sample with depression diagnosis as the outcome, there was no association between depression and 25(OH)D level (data not shown). A decreased risk of suicide was found for African Americans compared to other racial groups (OR = 0.56, 95% C.I.  = 0.36 to 0.88; Table 2) as well as for subjects with an increased length of deployment (OR  = 0.96, 95% C.I.  = 0.94 to 0.98; Table 2). When we excluded these covariates from the conditional regression model, we still found a significantly decreased risk of suicide for subjects with 25(OH)D concentrations falling in the 4^th^ octile (OR  = 0.34, 95% C.I.  = 0.19 to 0.61; Table 3). However, the model without covariates exhibits decreased goodness of fit [as measured by a higher Akaike Information Criterion (AIC) value] compared to the model with covariates (without covariates: AIC  = 681.45; with covariates: AIC = 651.25). DD Form 2796 includes the question “Over the last 2 weeks, how often have you been bothered by…“Thoughts that you would be better off dead or hurting yourself in some way”. This was endorsed by only 2 of 300 controls (0.7%) and 7 of 300 suicide cases (2.3%), too small a percentage for this to be analyzed systematically.

**Table 3:** Results of the conditional logistic regression analysis not including covariates.

Parameter	No. Cases/ Controls	DF	Estimate	Standard Error	Wald Chi-Square	P value	Odds Ratio^4^ (95% Conf. Limits)
Vitamin D Octile 1^1^	84/61		reference				
Vitamin D Octile 2	58/62	1	−0.42	0.26	2.6	0.11	0.66 (0.40, 1.10)
Vitamin D Octile 3	56/63	1	−0.5	0.26	3.7	0.05	0.61 (0.37, 1.01)
Vitamin D Octile 4	31/60	1	−1.07	0.3	13.23	0.0003	0.34 (0.19, 0.61)
Vitamin D Octile 5	55/62	1	−0.49	0.26	3.47	0.07	0.61 (0.37, 1.03)
Vitamin D Octile 6	85/65	1	−0.05	0.25	0.04	0.85	0.95 (0.58, 1.56)
Vitamin D Octile 7	67/59	1	−0.23	0.25	0.81	0.37	0.80 (0.48, 1.31)
Vitamin D Octile 8	59/63	1	−0.4	0.26	2.33	0.13	0.67 (0.40, 1.12)

1 Octiles were calculated from waveform adjusted 25(OH)D levels based on the distribution of the control subjects. Cutoff 25-hydroxyvitamin D values were as follows. octile 1: ≤15.5; octile 2: 15.6–18.8; octile 3: 18.9–21.2; octile 4: 21.3–22.9; octile 5: 23.0–25.4; octile 6: 25.5–29.2; octile 7: 29.3–36.0; octile 8: >36.0.

## Discussion

To our knowledge, this is the first study to examine the relationship between vitamin D status and suicide risk. We found that the risk for suicide was increased in the lowest octile of 25(OH)D levels, all the members of which had seasonally adjusted levels of 25(OH)D below 20 ng/mL. When the odds ratios for each octile are fitted to a decreasing exponential function (Fig 1), the result is a curve that is characteristic of nutrients, which typically manifest their specific effects only up to some threshold intake level and above which additional intake confers no further benefit [16] Once the nutrient response threshold has been reached, there is no dose response relationship observed, i.e. if there is sufficient 25(OH)D to prevent some heath outcome, further increase in the levels of 25(OH)D will have no effect on this outcome [17]. The excess risk of suicide in the lowest 25(OH)D octile translates to ∼25 deaths out of the octile total of 84. This is about 30% of the suicides in this octile (or 5% for the total of all octiles). Although problems with measurement of 25(OH)D can complicate the comparison between studies [3], our values for 25(OH)D provide a reasonable estimate of the vitamin D status of active duty service members, as they are roughly comparable to other studies of the military [18]. Vitamin D deficiency is often defined as a level of 25(OH)D below 20 ng/ml [4], a level not always associated with clinically evident symptoms, but rather with histological evidence of osteomalacia [3]. Based on this criterion and these issues, our finding that more than 30% of active duty personnel had 25(OH)D levels below 20 ng/ml is cause for concern.

Our results constitute one of the largest studies of vitamin D in the military. However, because we were unable to match controls and suicide cases by the exact date of blood collection, the season of blood collection was not balanced between groups; more controls had their blood drawn in the spring when 25(OH)D is lowest, and more suicide cases had their blood drawn in the fall when 25(OH)D is highest. Not surprisingly, seasonal adjustment was critical for proper analysis of our data, as 25(OH)D levels averaged the same in both groups before adjustment. The date of blood draw was unrelated to the date of suicide suggesting that the imbalance in the season of blood draw was due to chance. One limitation of our study is that 25(OH)D was not measured at the time of the suicide, but some months earlier; other studies of this population have used similar methods [19]. The significance of this issue is lessened, however, by reports of a strong correlation (r = 0.7) between 25(OH)D values that were measured three years apart [20]. This lack of intra-individual variation can at least be partially explained by the critical influence of genetic factors on vitamin D status [21].

Our findings that African Americans had lower levels of 25(OH)D and also lower rates of suicide is consistent with other studies [3], [22]. Because we controlled for race in our analysis, any compensatory mechanisms that may exist for such individuals were unlikely to confound our results. A history of depression, on the other hand, is a potential confound, as depressed individuals might spend more time indoors, reducing the production of vitamin D by sunlight. We addressed this confound by only including subjects that were on active duty, and by including depression diagnosis as a covariate. Furthermore, we detected no relationship between 25(OH)D levels and a diagnosis of depression in a follow up analysis. The shorter length of deployment noted among suicides is not surprising, as mental health stressors may precipitate a shortened deployment. Importantly, the distribution of increased suicide risk suggesting a protective threshold value of 25(OH)D is uncharacteristic of a confounding factor. Indeed, if a confounder were responsible for an association between suicide and low 25(OH)D, lower average adjusted 25(OH)D levels would be expected in the cases compared to the controls, but they were not. Instead, there was an increased risk for suicide in only the lowest octile of 25(OH)D. It is unlikely that a confounding factor would act only at the very lowest levels of 25(OH)D.

Our findings are observational in character, and hence do not establish a causal role for vitamin D deficiency and suicide. It is possible that sunlight may exert beneficial effects that are independent of vitamin D, as suggested by the fact that light therapy can reduce suicidal ideation in patients with seasonal affective disorder [23]. Vitamin D status may correspond with seasonal changes in the sunlight induced suppression of melatonin, and this may also be related to seasonal changes in the incidence of suicide [24]. However, a number of factors support the possibility that vitamin D may have at least some causal role in neuropsychiatric disorders such as suicide. In recent studies, low vitamin D status has been associated with reduced cognitive performance [25], psychotic-like symptoms [26], and the subsequent development of depression [6]. One study found an inverse association between dietary vitamin D and depression [7], and a number of clinical trials have shown positive effects of vitamin D on mood and depression [8], although other studies report no such effect [3].

Psychiatric illness is usually diagnosable retrospectively after suicides [22], and depressive illness is considered a major risk factor for suicide. However, more than 13% of our control subjects had a prior history of depression, compared to just 22% of the suicide cases. As depressive illness does not always precede suicide and was not associated with 25(OH)D in our sample [22], this leads us to other considerations. The development of suicidal ideation can be sudden, as the first thoughts of suicide often occur less than 10 minutes before it is attempted [27]. It may be that in a young, sometimes aggressive population such as the military, impulsivity plays more of a role than depression in risk for suicide. A potential link between vitamin D deficiency and impulsivity is suggested by findings that vitamin D deficiency may increase brain inflammatory cytokines, which can reduce serotonergic activity [28], and which have been associated with suicide,[5], [29]. Considering that reduced serotonergic activity has been associated with impulsive suicide [22], this could also explain why suicide and low serotonin are more prevalent in the spring [2], [3], [30].

Military service requirements for protective clothing and night time operations may reduce the opportunity for normal sunlight exposure. In a recent study, 25(OH)D levels fell in new recruits after eight weeks of combat training in South Carolina, even though it was summer [18]. Although our study does not provide a causal link between low vitamin D status and suicide, it does show that many military service members have inadequate levels of 25(OH) D levels. Our findings suggest that eliminating vitamin D deficiency to address more established vitamin D related issues in the military, e.g. stress fractures, might also have the additional benefit of reducing the risk of suicide [4], [31]. Studies are urgently needed to develop an appropriate strategy to insure that service members do not suffer ill effects from a preventable deficiency of vitamin D.

## References

[1] Preti A (1998) The influence of climate on suicidal behaviour in Italy. Psychiatry Res 78: 9–19. 9579698 10.1016/s0165-1781(97)00154-6

[2] Postolache TT, Mortensen PB, Tonelli LH, Jiao X, Frangakis C, et al (2010) Seasonal spring peaks of suicide in victims with and without prior history of hospitalization for mood disorders. J Affect Disord 121: 88–93. 19535151 10.1016/j.jad.2009.05.015 PMC2837087

[3] Holick MF, SpringerLink (2010) Vitamin D: physiology, molecular biology, and clinical applications: Humana.

[4] Holick MF, Binkley NC, Bischoff-Ferrari HA, Gordon CM, Hanley DA, et al (2011) Evaluation, treatment, and prevention of vitamin D deficiency: an Endocrine Society clinical practice guideline. J Clin Endocrinol Metab 96: 1911–1930. 21646368 10.1210/jc.2011-0385

[5] McCann JC, Ames BN (2008) Is there convincing biological or behavioral evidence linking vitamin D deficiency to brain dysfunction? FASEB J 22: 982–1001. 18056830 10.1096/fj.07-9326rev

[6] May HT, Bair TL, Lappe DL, Anderson JL, Horne BD, et al (2010) Association of vitamin D levels with incident depression among a general cardiovascular population. Am Heart J 159: 1037–1043. 20569717 10.1016/j.ahj.2010.03.017

[7] Bertone-Johnson ER, Powers SI, Spangler L, Brunner RL, Michael YL, et al (2011) Vitamin D intake from foods and supplements and depressive symptoms in a diverse population of older women. Am J Clin Nutr 94: 1104–1112. 21865327 10.3945/ajcn.111.017384 PMC3173027

[8] Jorde R, Sneve M, Figenschau Y, Svartberg J, Waterloo K (2008) Effects of vitamin D supplementation on symptoms of depression in overweight and obese subjects: randomized double blind trial. J Intern Med 264: 599–609. 18793245 10.1111/j.1365-2796.2008.02008.x

[9] Kuningas M, Mooijaart SP, Jolles J, Slagboom PE, Westendorp RG, et al (2009) VDR gene variants associate with cognitive function and depressive symptoms in old age. Neurobiol Aging 30: 466–473. 17714831 10.1016/j.neurobiolaging.2007.07.001

[10] Kjaergaard M, Joakimsen R, Jorde R (2011) Low serum 25-hydroxyvitamin D levels are associated with depression in an adult Norwegian population. Psychiatry Res 190: 221–225. 21784535 10.1016/j.psychres.2011.06.024

[11] Hoang MT, Defina LF, Willis BL, Leonard DS, Weiner MF, et al (2011) Association between low serum 25-hydroxyvitamin D and depression in a large sample of healthy adults: the Cooper Center longitudinal study. Mayo Clin Proc 86: 1050–1055. 22033249 10.4065/mcp.2011.0208 PMC3202994

[12] Jaddou HY, Batieha AM, Khader YS, Kanaan SH, El-Khateeb MS, et al.. (2011) Depression is associated with low levels of 25-hydroxyvitamin D among Jordanian adults: results from a national population survey. Eur Arch Psychiatry Clin Neurosci. 10.1007/s00406-011-0265-8 21993566

[13] Lewis MD, Hibbeln JR, Johnson JE, Lin YH, Hyun DY, et al.. (2011) Suicide deaths of active-duty US military and omega-3 fatty-acid status: a case-control comparison. J Clin Psychiatry. 10.4088/JCP.11m06879 PMC3259251 21903029

[14] Lappe JM, Davies KM, Travers-Gustafson D, Heaney RP (2006) Vitamin D status in a rural postmenopausal female population. J Am Coll Nutr 25: 395–402. 17031008 10.1080/07315724.2006.10719551

[15] Wang Y, Jacobs EJ, McCullough ML, Rodriguez C, Thun MJ, et al (2009) Comparing methods for accounting for seasonal variability in a biomarker when only a single sample is available: insights from simulations based on serum 25–hydroxyvitamin d. Am J Epidemiol 170: 88–94. 19406919 10.1093/aje/kwp086

[16] Heaney RP, Davies KM, Chen TC, Holick MF, Barger-Lux MJ (2003) Human serum 25-hydroxycholecalciferol response to extended oral dosing with cholecalciferol. Am J Clin Nutr 77: 204–210. 12499343 10.1093/ajcn/77.1.204

[17] Heaney RP (2012) Vitamin D-Baseline Status and Effective Dose-Editorial. N Engl J Med. 10.1056/NEJMe1206858 22762324

[18] Andersen NE, Karl JP, Cable SJ, Williams KW, Rood JC, et al (2010) Vitamin D status in female military personnel during combat training. J Int Soc Sports Nutr 7: 38. 21156069 10.1186/1550-2783-7-38 PMC3017021

[19] Munger KL, Levin LI, Hollis BW, Howard NS, Ascherio A (2006) Serum 25-hydroxyvitamin D levels and risk of multiple sclerosis. Jama 296: 2832–2838. 17179460 10.1001/jama.296.23.2832

[20] Platz EA, Leitzmann MF, Hollis BW, Willett WC, Giovannucci E (2004) Plasma 1, 25-dihydroxy-and 25-hydroxyvitamin D and subsequent risk of prostate cancer. Cancer Causes and Control 15: 255–265. 15090720 10.1023/B:CACO.0000024245.24880.8a

[21] Snellman G, Melhus H, Gedeborg R, Olofsson S, Wolk A, et al (2009) Seasonal genetic influence on serum 25-hydroxyvitamin D levels: a twin study. PloS one 4: e7747. 19915719 10.1371/journal.pone.0007747 PMC2774516

[22] Mann JJ (2003) Neurobiology of suicidal behaviour. Nat Rev Neurosci 4: 819–828. 14523381 10.1038/nrn1220

[23] Lam RW, Tam EM, Shiah IS, Yatham LN, Zis AP (2000) Effects of light therapy on suicidal ideation in patients with winter depression. J Clin Psychiatry 61: 30–32. 10695643 10.4088/jcp.v61n0108

[24] Havaki-Kontaxaki BJ, Papalias E, Kontaxaki ME, Papadimitriou GN (2010) Seasonality, suicidality and melatonin. Psychiatrike 21: 324–331. 21914615

[25] Annweiler C, Schott AM, Allali G, Bridenbaugh SA, Kressig RW, et al (2010) Association of vitamin D deficiency with cognitive impairment in older women: cross-sectional study. Neurology 74: 27–32. 19794127 10.1212/WNL.0b013e3181beecd3

[26] Hedelin M, Lof M, Olsson M, Lewander T, Nilsson B, et al (2010) Dietary intake of fish, omega-3, omega-6 polyunsaturated fatty acids and vitamin D and the prevalence of psychotic-like symptoms in a cohort of 33,000 women from the general population. BMC Psychiatry 10: 38. 20504323 10.1186/1471-244X-10-38 PMC2889879

[27] Deisenhammer EA, Ing CM, Strauss R, Kemmler G, Hinterhuber H, et al (2009) The duration of the suicidal process: how much time is left for intervention between consideration and accomplishment of a suicide attempt? J Clin Psychiatry 70: 19–24. 19026258

[28] Dantzer R, O'Connor JC, Lawson MA, Kelley KW (2011) Inflammation-associated depression: From serotonin to kynurenine. Psychoneuroendocrinology 36: 426–436. 21041030 10.1016/j.psyneuen.2010.09.012 PMC3053088

[29] Lindqvist D, Janelidze S, Erhardt S, Traskman-Bendz L, Engstrom G, et al (2010) CSF biomarkers in suicide attempters–a principal component analysis. Acta Psychiatr Scand 124: 52–61. 21198458 10.1111/j.1600-0447.2010.01655.x

[30] Brewerton TD, Berrettini WH, Nurnberger JI Jr, Linnoila M (1988) Analysis of seasonal fluctuations of CSF monoamine metabolites and neuropeptides in normal controls: findings with 5HIAA and HVA. Psychiatry Res 23: 257–265. 2455302 10.1016/0165-1781(88)90016-9

[31] Lappe J, Cullen D, Haynatzki G, Recker R, Ahlf R, et al (2008) Calcium and vitamin d supplementation decreases incidence of stress fractures in female navy recruits. J Bone Miner Res 23: 741–749. 18433305 10.1359/jbmr.080102

