# Correction: Low Vitamin D Status and Suicide: A Case-Control Study of Active Duty Military Service Members

**DOI:** 10.1371/annotation/9af84cbe-5576-4c4b-871c-f7ab0c64b9fd

**Published:** 2013-09-19

**Authors:** John C. Umhau, David T. George, Robert P. Heaney, Michael D. Lewis, Robert J. Ursano, Markus Heilig, Joseph R. Hibbeln, Melanie L. Schwandt

Under the "Characteristic" column of Table 1, the units for 25-hydroxyvitamin D^1,2^ (SEM) are listed as g/ml. It should read "25-hydroxyvitamin D^1,2^ (SEM), ng/ml" 

